# Siamese Network-Based All-Purpose-Tracker, a Model-Free Deep Learning Tool for Animal Behavioral Tracking

**DOI:** 10.3389/fnbeh.2022.759943

**Published:** 2022-03-04

**Authors:** Lihui Su, Wenyao Wang, Kaiwen Sheng, Xiaofei Liu, Kai Du, Yonghong Tian, Lei Ma

**Affiliations:** ^1^School of Computer Science, Peking University, Beijing, China; ^2^Beijing Academy of Artificial Intelligence, Beijing, China; ^3^Institute for Artificial Intelligence, Peking University, Beijing, China; ^4^Peng Cheng Laboratory, Shenzhen, China

**Keywords:** behavioral tracking, deep learning, model-free, universality, Siamese network

## Abstract

Accurate tracking is the basis of behavioral analysis, an important research method in neuroscience and many other fields. However, the currently available tracking methods have limitations. Traditional computer vision methods have problems in complex environments, and deep learning methods are hard to be applied universally due to the requirement of laborious annotations. To address the trade-off between accuracy and universality, we developed an easy-to-use tracking tool, Siamese Network-based All-Purpose Tracker (SNAP-Tracker), a model-free tracking software built on the Siamese network. The pretrained Siamese network offers SNAP-Tracker a remarkable feature extraction ability to keep tracking accuracy, and the model-free design makes it usable directly before laborious annotations and network refinement. SNAP-Tracker provides a “tracking with detection” mode to track longer videos with an additional detection module. We demonstrate the stability of SNAP-Tracker through different experimental conditions and different tracking tasks. In short, SNAP-Tracker provides a general solution to behavioral tracking without compromising accuracy. For the user’s convenience, we have integrated the tool into a tidy graphic user interface and opened the source code for downloading and using (https://github.com/slh0302/SNAP).

## Introduction

Living organisms receive cues from external environments, process the information internally, and finally output the processing outcomes in the form of behavior. Therefore quantitatively modeling and analyzing behavior is vital to help understand the motivations and underlying mechanisms of animals and is thus widely used in neuroscience (Frye and Dickinson, 2004; Krakauer et al., 2017) and other animal-related disciplines, such as psychology (Snowdon, 1983; Dewsbury, 1992), ecology (Nathan et al., 2008; Dall et al., 2012). The recent decades have witnessed the application of technology in recording and observing animal behavior, which has greatly liberated human labor in behavioral data acquisition, and yielded large amounts of data with unprecedented spatial and temporal resolutions (Gomez-Marin et al., 2014). These explosive animal behavioral data bring significant challenges to analysis. Fortunately, automated image-based processing methods offer opportunities to solve the challenges in behavioral analysis (Dell et al., 2014) and open up a new field called computational ethology that aims to quantify animal behavior (Anderson and Perona, 2014). Accurate trajectory tracking is the first and most crucial step of behavioral analysis (Pereira et al., 2020).

Recent advances in computer vision (CV) and deep learning have inspired many well-behaved tracking methods. Among different algorithms developed on traditional CV techniques, background subtraction is the earliest and most commonly used by software such as ToxTrac (Rodriguez et al., 2017). There are also software packages that apply other efficient object segmentation methods, such as the adaptive thresholding in Tracktor (Sridhar et al., 2019). To track individuals in groups, which can be disturbed by the touching and crossing among individuals, idTracker (Pérez-Escudero et al., 2014) uses regressive features of all individuals and successfully tracks multiple individuals simultaneously. The above-mentioned methods have shown successful tracking performance in particular conditions. However, they still have some limitations, of which the most critical one is that these methods work fine only in constrained environments because of the relatively simple features extracted by their segmentation methods. Deep learning, which is the most popular method in image processing (LeCun et al., 2015), has provided significant breakthroughs in designing video-based animal behavior tracking algorithms (Mathis and Mathis, 2020). Representative examples are idTracker.ai (Romero-Ferrero et al., 2019) for multiple individual tracking and DeepLabCut (DLC; Mathis et al., 2018), LEAP (Pereira et al., 2019), and DeepPoseKit (Graving et al., 2019) for high-dimensional postures tracking. The outstanding feature extraction ability of deep learning significantly improves the performance of tracking tools in complex environments. However, a common problem for both traditional and deep learning methods is their performance loss in “open” conditions, in which the statistical distributions of test datasets are different from those of training datasets (Goodfellow et al., 2014; Nguyen et al., 2015). The most effective solution for deep learning methods is enough training samples. Thus when applying deep learning methods in practice, researchers have to manually annotate a certain number of video frames to collect enough training samples, a well-known difficult task in biological fields requiring expertise and time. Besides, researchers should also be equipped with professional knowledge to train or fine-tune the neural networks. Therefore, solving practical problems by taking advantage of deep learning while bypassing its overdependence on data is a hot topic in the deep-learning field. We think SNAP-Tracker is a successful attempt to implement this idea in animal behavioral tracking.

To alleviate the burden of researchers and promote the development of behavioral analysis, in this article, we present an accurate, universal, and easy-to-use tracking software, Siamese Network based All-Purpose Tracker (SNAP-Tracker). As its name suggests, we develop SNAP-Tracker upon a pretrained Siamese network, consisting of two identical subnetworks to extract features and make comparisons (Bromley et al., 1993). Although developed upon deep learning methods, SNAP-Tracker works in a model-free way to track the object without premodeling it first. Thus, it no longer requires refinements after network pretraining. A region-of-interest (ROI) align and distractor learning protocol has been applied to the Siamese network to help overcome the disturbance from background information (Su et al., 2020). Briefly, the ROI-aligned operation can promise a smaller data loss/data gain ratio than the ordinary ROI pooling operation, so it is set before ROI pooling in the template branch to generate more accurate target features. SNAP-Tracker’s graphic user interface (GUI) is tidy and easy to operate (Supplementary Figure 1). In most cases, users only need to define the tracking target with a bounding box at the beginning of the videos, just like taking a “snapshot” of the target, and SNAP-Tracker will use the “snapshot” as the beginning template to finish the following tracking procedure. Experimental results displayed that SNAP-Tracker can accomplish tracking tasks across various species and environmental conditions without compromising performance. With an additional detection module, SNAP-Tracker can behave in the “tracking with detection” mode, suitable for dealing with larger datasets or more complicated tracking tasks. However, different from other “tracking by detection” software, the detection module of SNAP-Tracker is only activated when tracking failures might happen, which can improve the overall accuracy but will not affect processing speed too much. To sum up, with SNAP-Tracker, accurate tracking, can become more accessible and more efficient.

## Materials and Methods

### Datasets

#### Mouse Freely Running Dataset

The dataset describes the freely running behavior of mice with their heads fixed. It consists of seven raw videos, provided by Jun Ding’s Lab from Stanford University. The videos were captured from the side, and each one recorded 5,000 frames (896 × 600 pixels) for about 3 min. All experimental procedures were conducted in accordance with protocols approved by Stanford University’s Administrative Panel on Laboratory Animal Care. We separated the seven videos into four groups according to foot illumination, roller color, and head direction (Table 1). Throughout all the seven videos, the forefoot and hindfoot on the closer side to the camera were manually annotated with bounding boxes and served as the ground truth to test the performance of the tracking tools. The dataset is available at https://drive.google.com/file/d/1k0w_lgIBd5xIY0f63J8VfuccvHZ7spsD/view?usp=sharing.

**TABLE 1:** Groups of the mouse freely running dataset.

Groups	Video characteristics			Examples
	Foot illumination	Roller color	Head direction	
1	No	Dark	Left	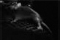
2	Yes	Dark	Left	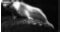
3	No	Light	Left	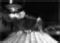
4	No	Light	Right	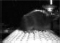

#### Other Datasets

The zebrafish dataset is a video of five freely swimming zebrafish recorded from the top. An example video of idTracker is available from Perez-Escudero et al. (2014), and we downloaded it from http://www.idtracker.es/. The mouse pupil dataset displays the abnormal pupil constriction behavior in the absence of intrinsic photosensitive retinal ganglion cells glutamate (Keenan et al., 2016). The chimpanzee dataset is a video about the tapping behavior of a chimpanzee on a keyboard we downloaded from Hattori et al. (2013). The peacock spider dataset displays the courtship body behavior of peacock spiders (Girard et al., 2011). The blue-capped cordon-bleu dataset records the multimodal courtship of birds (Ota et al., 2015).

### Siamese Network-Based All-Purpose-Tracker

#### Overview

Siamese Network-based All-Purpose-Tracker was written in Python 3 and implemented with PyTorch 0.4.0. We develop the GUI with Qt 5.13.0. We have tested its availability on Ubuntu 16.04 and Windows 10. More detailed information, including the executable file, the master code, and others, can be found in the GitHub repository: https://github.com/slh0302/SNAP.

The basic structure of SNAP-Tracker follows the framework of the Siamese network (Bertinetto et al., 2016), which consists of twin-deep convolution networks sharing the same set of parameters. The first essential module of SNAP-Tracker is the feature extractor pretrained on ImageNet, which can be either a 5-layer AlexNet (Krizhevsky et al., 2012) or a 50-layer ResNet-50 (He et al., 2016). Experiments in this paper were all performed with the faster AlexNet. Another essential module is the similarity metric module used for calculating the cross-correlation between the template and target frames. The area with maximum similarity will be decided as the tracking location. To decrease the disturbance of background information, we have developed an ROI align and distractor learning protocol (Su et al., 2020). Briefly speaking, the ROI align layer is placed after the feature extractor to maintain the template scale with more accurate features and exclude the disturbance from marginal background information. Further distractor learning is performed after cross-correlation calculation to increase the Euclidian distance between the target and distractors.

We have also implemented a “tracking with detection” mode with an additional detection module, a Faster-RCNN with a 50-layer ResNet that can be trained with the primary tracking results offered by the basic tracking module. When the detection module is activated, if the output confidence of SNAP-Tracker is below 0.3, it will help guide the tracking procedure.

#### Network Training

We have used AlexNet (Krizhevsky et al., 2012) and stride-reduced ResNet50 (He et al., 2016) as the backbone network to perform proposal classification and bounding box regression with five anchors as in Li et al. (2018). The backbone network of our architecture was pretrained on ImageNet (Russakovsky et al., 2015). Then we further trained the whole neural network of SNAP-Tracker on COCO (Lin et al., 2014), ImageNet DET (Deng et al., 2009), ImageNet VID (Russakovsky et al., 2015), and YouTube-Bounding Boxes Dataset (Real et al., 2017) to learn a general measurement of similarities between objects for visual tracking. In both training and testing, we used single-scale images with 127 pixels for template patches and 255 pixels for searching regions. We applied stochastic gradient descent with the momentum of 0.9 and a weight attenuation of 0.0005 as the optimizing method. We warmed up ResNet50 with a learning rate of 0.005 for the first five epochs. For AlexNet, we fixed the parameters of its first three layers, and AlexNet did not need a warm-up at the beginning of the training. Then we set 0.001 and 0.0001 as the learning rate of the backbone network and the rest of the network (Zhu et al., 2018; Li et al., 2019). The learning rate decayed exponentially to one-tenth of the original value.

The detection module of SNAP-Tracker is a pretrained 50-layer ResNet-based Faster-RCNN. For the retraining of the detection module, we kept all of the parameters default. We collected 10–50K frames by the basic SNAP-Tracker for retraining, the initial learning rate was 0.001, and the batch size was 32. For DLC and LEAP, the initial learning rate was 0.005 and 0.0001, and the batch size was 16 and 8, correspondingly.

#### Output Confidence

Output confidence represents the confidence level of the model about results. It is used in the “tracking with detection” mode to activate the detection module. As we can regard object tracking as a binary classification problem between the tracking target and background information, we used the classification probability of the tracking target as the output confidence.

### Experimental Design

#### Evaluation Criteria

Overlap rate (OR) is the ratio of intersection area to union area between tracking results and human annotations. We used OR to evaluate the tracking accuracy of software using bound boxes as the tracking results. We set the threshold of success at 0.5. If the OR value of the result is higher than the threshold, we can consider that the tracking is successful, and the success rate means the ratio of successful frames. We ran a test on the first 1,300 frames of mouse freely running video 1 and found out that the bounding box size in the first frame could affect the final successful rate (Supplementary Figure 2); and in our results, we chose circumscribed rectangle as the bounding box size by our experience.

We also used pixel error (PE) to evaluate the accuracy of tracking software when the tracking results of the software are points. PE is the Euclidean distance between the tracking results of software and human annotations. Positions of tracking points or bounding boxes centers refer to the tracking results of the software. We set the successful threshold at 20 pixels. If the PE value is lower than the threshold, we can consider that successful tracking and the accuracy rate means the ratio of successful frames.

#### Experiments Description

To reveal that few human corrections are helpful to maintain high accuracy (Figure 2), feet tracking was performed on different continuous frames (up to 3,000) of three videos from the mouse freely running dataset (Table 1). We used OR as the evaluation criterion for calculating error rates, which were the ratios of the number of failing frames to total frames (Figure 2B). Label efforts under different tracking frames mean the ratio of human correcting frames to total frames (Figure 2C).

**FIGURE 1:**
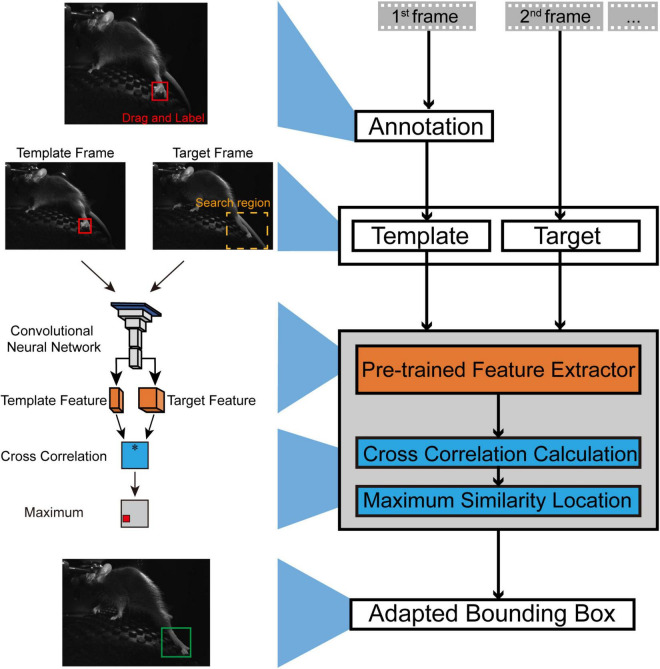
An illustration of the workflow for SNAP-Tracker. In the typical workflow of SNAP-Tracker, users should make the only annotation at the first frame of the video by dragging a bounding box out of the tracking object, which will serve as the template for the second and other later frames. In the search region of a target frame, the image feature is extracted by the pretrained feature extractor simultaneously with the template. After comparing the cross-correlation between the template and target frame feature, the similarity metric module will select the location with maximum similarity and generate an adapted bounding box outside the tracking object. Connecting the bounding boxes of all frames in series can form the object’s trajectory. * Denotes the similarity function (i.e., cross correlation) to be computed for target and template feature.

**FIGURE 2:**
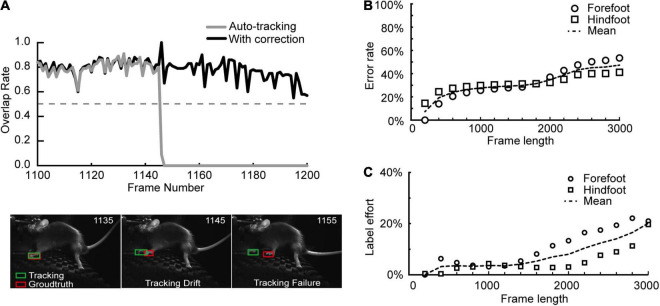
Few human corrections are helpful to increase accuracy. **(A)** In a practical auto-tracking procedure (the gray line in the top plot), the tracking result (the green box) can match the ground truth (the red box) most of the time (the bottom left inset). Tracking drift may occasionally happen due to the fast movement of the object or another similar object nearby (the bottom middle inset). The tracking drift can evolve to tracking failure without a correction (the bottom right inset). However, a single correction on the earliest tracking drift frame can successfully rescue the subsequent tracking failure (the black line in the top plot). *X*-axis: Frame number of the video; *Y*-axis: overlap rate with the evaluation threshold of 0.5. **(B)** The tracking error rates of forefoot and hindfoot increased with video frame length’s elongation. Error rate: the percent ratio of failed frames in the total frames. *N* = 3 videos. **(C)** Fewer human corrections than error frames are enough to fix the tracking failures and achieve 100% accurate tracking results. A 100% accuracy: all the frames’ OR values are above 0.5; label effort: the percent ratio of frames needs to be corrected to keep 100% accuracy. *N* = 3 videos.

**FIGURE 3:**
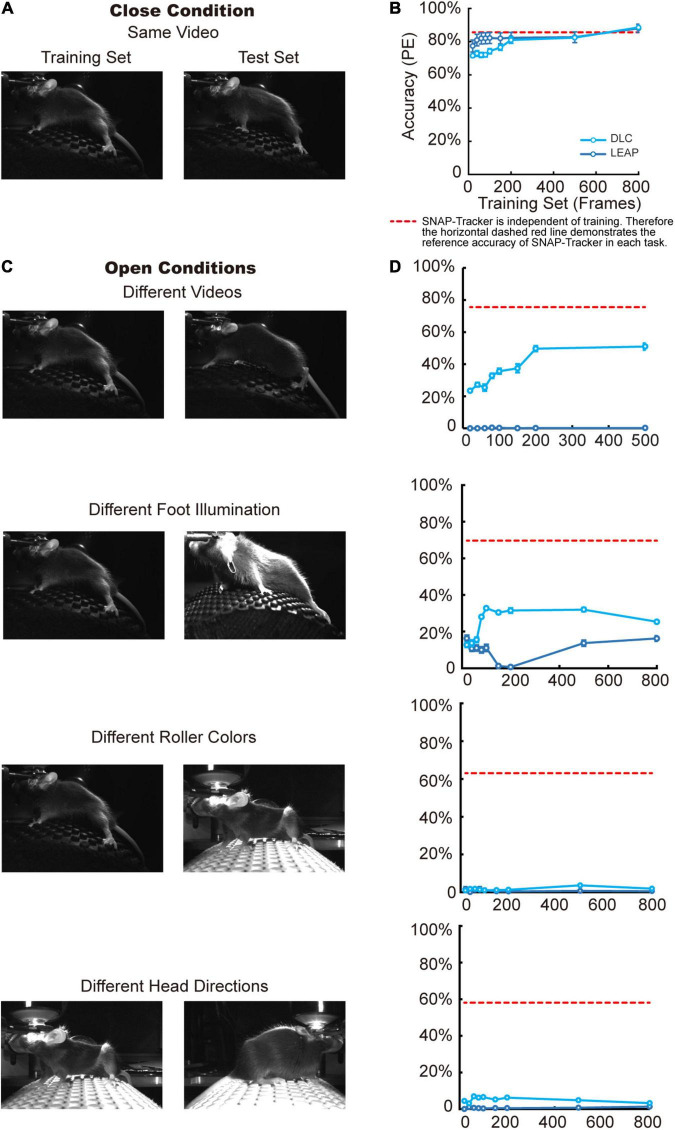
Stable performance of SNAP-Tracker in open conditions. **(A)** In the close condition, training and test frames come from the same video. **(B)** The accuracy of three methods in the close condition with different scales of the training set. The tracking accuracy of DLC (dare blue) and LEAP (light blue) increases with more training frames. SNAP-Tracker (red) is independent of training, and its performance is comparable to the other two. Accuracy: the ratio of frames with PE value lower than the threshold of 20 pixels. **(C)** In four kinds of open conditions, the test video can be a different one with similar environmental conditions (the first row) or has different illumination (the second row), different roller colors (the third row), different head directions (the last row). **(D)** In open conditions, the accuracies of DLC and LEAP both drop significantly even trained with the highest number of training frames. However, SNAP-Tracker can still keep relatively good performance due to its independence to training.

To illustrate the stability of SNAP-Tracker in open conditions, we used PE as the evaluation criterion to demonstrate the performance of SNAP-Tracker and two representative deep learning tracking tools, DLC (Mathis et al., 2018) and LEAP (Pereira et al., 2019; Figure 3). Test frames and training frames were from the same video in the close condition test, while from different videos or under different conditions in the open condition tests. We fixed the length of the testing frames at 1,000 and repeated each test session with randomly selected clips three times.

**FIGURE 4:**
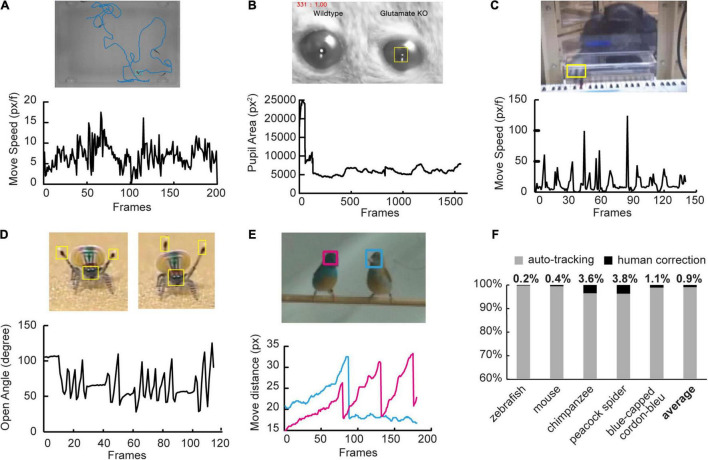
Broader applicability of SNAP-Tracker. **(A)** Top: In the case of individual tracking among five zebrafish, the cyan box indicates the target fish, and the blue line shows its moving trajectory. Bottom: The plot shows the variation of swimming speed (in pixel per frame) of the zebrafish. **(B)** Top: In the case of mouse pupil tracking, the box shows the pupil of a glutamate knockout mouse. Bottom: The plot reveals the variation of pupil area (in pixel^2^). **(C)** Top: In tracking the chimpanzee finger with complex background information, it is easy to locate the finger position accurately. Bottom: Tapping behavior can be observed in the movement speed plot (in pixel per frame). **(D)** Top: When analyzing the courtship behavior of a peacock spider, we tracked the tips of the pair of third legs and head. Bottom: The open angle between two third legs, a sign of the “Fan” dance, is speculated. **(E)** Top: In tracking two blue-capped cordon-blues, we tracked the positions of their heads with different color boxes. Bottom: The plot represents the movement of heads with corresponding colors, which exhibits the interactive bobbing behaviors of birds. **(F)** The bar plot shows the ratios of human correction in tasks. Percentages of human correction (black) in the five tasks are 0.2, 0.4, 3.6, 3.8, and 1.1% respectively. The average human correction is 0.9 ± 1.7%.

To test the applicability of SNAP-Tracker in broader situations, we used five other videos coming from published data (Figure 4). We validated and converted the tracking results to other indexes for further analysis, such as moving distance in pixel (px), moving speed in pixel per frame (px/f), area size in pixel square (px^2^), and angle in degree. We also recorded the times needed for human correction and exhibited it in the percentage of total frames.

**FIGURE 5:**
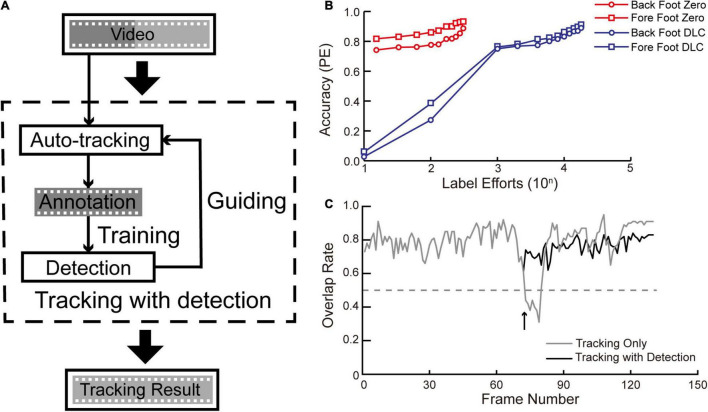
Tracking with detection mode. **(A)** In the tracking with detection mode, a video can be tracked by the auto-tracking mode first, as described above. Then the detection module can be trained by these annotation results and help improve the automatic tracking with higher accuracy. Iteratively, it is possible to track a long video stably with the well-trained detection module. We set the threshold at 0.3 for output confidence activating the detection module to avoid compromising processing speed. **(B)** The comparison of labeling efforts between the tracking with detection mode of SNAP-Tracker (red) and DLC (blue). The accuracy of DLC is positively related to the number of training images, which requires manual annotation. While for the detection module of SNAP-Tracker, the primary tracking module can provide most annotations, achieving similar accuracy with fewer human laborious by almost two magnitudes. Accuracy: the ratio of frames with PE value lower than the threshold of 20 pixels. **(C)** An example of autocorrection by the detection module. The tracking with detection mode can (the black line) prevent failures that happen in the tracking-only mode (the gray line). *X*-axis: Frame number of the video; *Y*-axis: overlap rate with the evaluation threshold of 0.5.

To compare the efficiency of the “tracking with detection” mode of SNAP-Tracker with other “tracking by detection” methods, such as DLC, we applied PE as the evaluation criterion to evaluate their success rates under different training frames (Figure 5B). In this experiment, we used all the seven mouse running videos as a whole to test both packages. We randomly selected 60% of the seven video frames to constitute the whole training set and evenly used 2–100% in the training sessions. For the working pipeline of DLC, training frames were precisely the handed-labeled ground truth annotations. So the label efforts equaled its training samples. For the working pipeline of the detection mode of SNAP-Tracker, the training frames were from its immediate automatic tracking results and occasional human corrections; and we took the human corrections as the label efforts it needed.

## Results

### Framework and Workflow of Siamese Network-Based All-Purpose-Tracker

We developed SNAP-Tracker on a deep Siamese neural network, one of the deep neural networks widely used in visual tracking. SNAP-Tracker is a model-free tracker and thus can complete tracking tasks without modeling the object priorly, different from other model-based deep learning methods. There are two critical compositions in the basic framework of SNAP-Tracker (Figure 1). The feature extraction module (the orange part in Figure 1) is thoroughly pretrained first and then used for feature extraction from the bounding box of the template frame and the searching areas in target frames. The similarity metric module (the blue part in Figure 1) determines the object’s location by calculating the cross-correlation between the extracted features from the template and the target frames. To start a realistic tracking procedure, users can label the object in the first frame as the template and then give hands to SNAP-Tracker, which will automatically label-size adaptive bounding boxes outside the tracking object according to the maximum feature similarity to the template. With the sliding of video frames, SNAP-Tracker annotates each frame continuously and finally produces the trajectory of the tracking object through the video. Supplementary Video 1 shows a practical case of the workflow, which is easy to operate. As described above, throughout the whole tracking procedure, usually the only thing users have to do is define their interested tracking objects at the first frame with a bounding box and then handing over the task to SNAP-Tracker by simply clicking the starting button in the GUI (Supplementary Figure 1).

### Few Human Corrections Are Helpful to Keep High Accuracy

Tracking failure is a common problem for tracking tools, which can happen when the tracking object moves too fast or when another similar object occurs nearby. In these cases, the software would accumulate errors without human interference. Therefore we integrated a manually auxiliary correction module into the basic operation panel of SNAP-Tracker (the dashed box E in Supplementary Figure 1). Users can rescue tracking failures by stopping the tracking and correcting the error with a new bounding box, which will change the original template into a new annotation of the current frame. After this, we can restart tracking from the breaking point (Supplementary Video 2 shows a practical case). We have shown the efficiency of human correction in preventing tracking errors with the first video of the mouse dataset (Figure 2A). In this experiment, we used OR as the evaluation criteria and set the threshold at 0.5; tracking failure meant the OR was below the threshold. The OR of the 1,145th frame dropped suddenly below the threshold of 0.5, indicating a tracking failure might happen, which was the tracking drift to the other forefoot (the bottom middle inset of Figure 2A). Without human correction, SNAP-Tracker regarded the wrong foot as the tracking object, and tracking failure could happen (the bottom right inset of Figure 2A). Sometimes, it was probable for SNAP-Tracker to automatically relocate the target if the correct foot appeared again in the searching region of SNAP-Tracker. However, if we could timely correct the shifted bounding box at the 1,145th frame where the tracking drift started, the continuous tracking failures could be avoided to a large degree. Intuitively, failures would increase with the length of the video being longer. To reveal that few human corrections are helpful to keep high accuracy in this situation, we performed feet tracking on the mouse dataset with different continuous frames clips (up to 3,000). As expected, error rates, indicating the ratio of failed tracking frames to total frames, increased with longer clips (Figure 2B). But a certain number of label efforts, representing the ratio of human correcting frames, were enough to keep the OR of each frame steady above the threshold, which we identified as 100% tracking accuracy (Figure 2C). It must be noted that the OR value and final successful/error rate can be influenced by the bounding box size, as we tested on the first 1,300 frames of the video used in Figure 2A (Supplementary Figure 2). Our paper used circumscribed rectangle as the bounding box covering the target while the size was as small as possible. In short, SNAP-Tracker can complete much better tracking with acceptable times of timely corrections by humans.

### Stable Performance of Siamese Network-Based All-Purpose-Tracker in Open Conditions

Siamese Network-based All-Purpose-Tracker is a model-free tracking package. Unlike the popular model-based methods, model-free methods do not need to learn the prior knowledge of the tracking object in advance. Therefore SNAP-Tracker does not require model retraining or parameters fine-tuning, which significantly alleviates the need for manual annotation before applied. The manual annotation needed in the first frame will not modify the model parameters but will tell SNAP-Track what the tracking target is. In this way, SNAP-Tracker can express much more stable performance in open conditions compared with other model-based tracking methods. To demonstrate the stable performance of SNAP-Tracker in open-conditions without enough training samples, we tested SNAP-Tracker and two other representative deep learning methods, DLC (Mathis et al., 2018) and LEAP (Pereira et al., 2019), on the mouse dataset (Figure 3). We used PE as the evaluation criterion of accuracy in this experiment and set the successful threshold at 20 pixels. The accuracy was the ratio of successful frames. When tested in close conditions, where the test dataset came from the same video as the training dataset (Figure 3A), SNAP-Tracker and the other two deep learning methods showed good performance (Figure 3B). It is worth noting that the two model-based deep learning methods displayed increasing accuracies with the increment of training data. However, the performance of SNAP-Tracker was independent of training due to the model-free tracking strategy, and we expressed its accuracy with a horizontal red dashed line in the figure for a better comparison. When it came to open conditions, test datasets had different feature distributions from the training dataset, such as different videos under similar environments or different videos with different conditions (foot illumination, roller colors, and head directions) (Figure 3C). It is evident in Figure 3D that the performance of model-based deep learning methods dropped sharply due to the lack of model fine-tuning with test data; only DLC did not show bad accuracy in the first situation in which the test dataset was the most similar to the training dataset. However, SNAP-Tracker could exhibit better and stable performance in different open conditions (Figure 3D). Furthermore, to clearly tell DLC and LEAP what to be tracked in the test video, we have added the first annotation of test videos, and the SNAP-Tracker was used for tracking, in each corresponding DLC and LEAP training session (Supplementary Figure 3). Compared with before (line with dots in Supplementary Figure 3), by training with one additional frame, the first annotation of test videos (smooth line in Supplementary Figure 3) could improve the accuracy but slightly. Therefore, SNAP-Tracker can be used directly with relatively stable performance, offering a choice for tasks with varying conditions.

### Broader Applicability of Siamese Network-Based All-Purpose-Tracker

Siamese Network-based All-Purpose-Tracker can have good applicability across different behavioral tracking paradigms. To demonstrate the broader applicability of SNAP-Tracker, we applied it in five other videos with different species and tasks coming from published data and made further analyses based on the primary tracking results (Figure 4). In this experiment, we used PE and the threshold of 20 pixels as the evaluation criterion of accuracy and defined the accuracy as the ratio of successful frames. We showed the averaged accuracy of 10 trails on each dataset (Supplementary Figure 4) and typical cases demonstrating the corresponding comparison with the ground truth (Supplementary Videos 3–7). In the individual tracking task of zebrafish, SNAP-Tracker can accurately track one of a collective of 5 zebrafish (the cyan bounding box and blue trajectory in Figure 4A), and we could obtain the swimming speed of the animal according to the tracking trajectory (Figure 4A). Besides individual tracking, tracking particular body parts of an animal, such as the contraction and dilation of pupils, is also essential in neuroscience. In the tracking of mouse pupils (Keenan et al., 2016), we could quickly identify the state of the pupil *via* the area of the inscribed ellipse of each bounding box, which could reveal the role of glutamate by comparing the difference between wild type and glutamate knockout mouse (Figure 4B). In another case of tracking the finger of a chimpanzee with a more complex background (Hattori et al., 2013), we could also get an accurate trace of the finger and infer the tapping frequency between alternative keys (Figure 4C). More than that, SNAP-Tracker could also be used for more sophisticated behavioral analysis, for example, the courtship behavior of peacock spiders (Girard et al., 2011) and blue-capped cordon-bleu (Ota et al., 2015). By tracking the pair of third legs and the head of a peacock spider, we could speculate the open angle between two third legs, which served as a constituent of “Fan” dance, a representative courtship posture of peacock spiders (Figure 4D). Similarly, by tracking the positions of the heads of two blue-capped cordon-bleu, we could extract out the interactive bobbing behavior between them from the video (Figure 4E). We recorded the number of corrections needed to keep 100% accuracy during tasks and found that none of the human corrections in five tasks was larger than 4% (Figure 4F). On average, human correction only occupied a tiny portion (0.9 ± 1.7% on average). Taken together, with a reasonable number of human corrections, users can apply SNAP-Tracker widely in various tracking tasks.

### Tracking With Detection Mode

As shown above, the need for human correction will increase with the elongation of tracking frames (Figure 2B). A strategy that can liberate human efforts is required in longer videos with more complex conditions. Therefore we developed a “tracking with detection” mode by providing SNAP-Tracker with an additional detection module. In its brief framework (Figure 5A), the automatic tracking results from the basic SNAP-Tracker serve as the training dataset for the detection module, and the detection module can help improve the accuracy of the basic auto-tracking module. After iterative training, the well-trained detection module can take the place of human correction when a tracking shift happens. Notably, the detection module only functions when the output confidence level of SNAP-Tracker is lower than the predefined threshold; users can set a higher threshold for better accuracy or a lower threshold for faster processing. In our experiment, we set the activation threshold at 0.3. A significant difference of the “tracking with detection” mode of SNAP-Tracker from DLC compared with a “tracking by detection” deep learning method is that SNAP-Tracker itself can offer tracking results as training data, saving much hand-labeling efforts. To demonstrate the efficiency of the “tracking with detection” mode of SNAP-Tracker, we tested the performance (with the criterion of PE) of SNAP-Tracker and another “tracking by detection” method with a synthetic free-running mouse video consisting of the seven videos (Figure 5B). We found that the “tracking with detection” mode can perform well with few label efforts (red lines in Figure 5B). However, the “tracking by detection” method (blue lines in Figure 5B) needed two magnitudes higher label efforts to achieve comparable accuracy. It should be clarified here that label effort has a different source in each method. Specifically, the label efforts of DLC equaled its training samples, while we took the human correction numbers as the label efforts for SNAP-Tracker. The “tracking with detection” mode can improve tracking efficiency compared with the basic SNAP-Tracker. In a typical tracking case of mouse foot, the “tracking with detection” mode (the black line in Figure 5C) could avoid tracking errors that happen under the regular mode (the gray line in Figure 5C). To sum up, the “tracking with detection” mode can replace the role of human intervention to complete more complex tracking tasks, which are suitable for dealing with larger datasets.

## Discussion

This article presents a model-free tracking software, SNAP-Tracker, which shows robust performance under various conditions. The software has already been pretrained with publicly available datasets and requires no more parameter fine-tuning when used in practical tasks, which greatly reduces the burden of users. Considering the user communities with different backgrounds, we have integrated the software into a compact and easy-to-use GUI. The “tracking with detection” mode of SNAP-Tracker is more automated with the help of a detection module, and we can apply it in more complex conditions. In a word, SNAP-Tracker can be a practical choice in different kinds of behavioral tracking analysis. We will discuss the characteristics of SNAP-Tracker from the following aspects.

### Benefits and Drawbacks of Deep Learning

The benefits of using deep learning in behavioral analysis are apparent as those in other CV fields. Compared to traditional CV methods, deep learning methods can achieve much more accurate performance at the human level and even beyond. So deep learning is the current trend in many fields, and popular tracking software packages use deep learning. Nevertheless, we should notice problems such as high computational consumption and overfitting in deep learning methods cautiously (Mathis and Mathis, 2020). Researchers have made contributions to decreasing training efforts and increasing processing speed. DLC (Mathis et al., 2018) was built in the way of transfer learning upon DeeperCut (Insafutdinov et al., 2016), a previously established model. Soon after, LEAP tried to improve the processing speed by applying a network with much fewer layers at the price of accuracy (Pereira et al., 2019). The more recent DeepPoseKit made considerable progress in both speed and robustness by using a multiscale deep-learning model (Graving et al., 2019). Even so, laborious annotations and network fine-tuning are inevitably needed, which can be much severe if the tracking task contains multiple individuals (Graving et al., 2019). With enough training samples, deep learning methods can perform very well. However, if there are not enough training samples, the performance of deep learning methods will be affected. The situation in practical neuroscience research could be much more challenging. The annotation of biological samples is a well-known arduous task, requiring expertise and much time. For example, when observing the courtship behavior of songbirds (Ota et al., 2015), scientists are interested in only a tiny portion of the whole video frames. Making annotation is time-consuming, and training in this few-shot situation is challenging. Another problem we have to resolve is the performance loss in “open” conditions. Environmental conditions, such as illumination, in practice can change during the task, but we cannot label a training set including all possibilities. In some particular tasks, such as screening mutant mice (Brown et al., 2000), the animal’s behavior is complex to be predefined. Thus, training in this situation will be a challenge. The idea of model-free tracking is a recently introduced solution to circumvent these drawbacks, which is the designing strategy of SNAP-Tracker.

### Model-Based and Model-Free Tracking

The model-based and model-free dichotomy is familiar in the CV tracking field. Although idTracker (Pérez-Escudero et al., 2014), idTracker.ai (Romero-Ferrero et al., 2019) were used in individual tracking, and DLC (Mathis et al., 2018), LEAP (Pereira et al., 2019), and DeepPosekit (Graving et al., 2019) were designed for pose estimation, all of them and many other tracking tools in ethology belong to model-based tracking (Worrall et al., 1991), which require prior knowledge of the objects before tracking. For model-based tracking, targets in the frames of a video are detected first by object detection or segmentation methods and then connected along the temporal series to generate the moving trajectory. We call this pipeline “tracking by detection”; the strategy of tracking by detection can increase tracking accuracy, but at the cost of processing speed and generalization. Differently, model-free tracking (Zhang and Van Der Maaten, 2013) is independent of the target’s prior modeling, and users can apply the method directly to broader tasks. Without premodeling, users can define the tracking target’s template in the first frame and then let the software finish tracking to the end frame by frame. In this way, SNAP-Tracker can be a universal method suitable for various behavioral missions.

### Individual Tracking and Pose Tracking

According to the analyzing resolution, we can classify behavioral analysis into different stages, from coarse to fine (Pereira et al., 2020), which can be summarized into two classes, individual tracking and pose tracking. They are the critical consideration for users to decide the options of tracking tools. In general, the spatiotemporal trajectory of single or multiple individuals is enough to answer questions (Berdahl et al., 2013; Mersch et al., 2013; Seibenhener and Wooten, 2015). To simplify the tracking of multiple individuals in a group, researchers usually labeled the targets with artificial markers (Ohayon et al., 2013; Shemesh et al., 2013), which might potentially affect animal behaviors (Dennis et al., 2008). By defining the model of each individual, idTracker (Pérez-Escudero et al., 2014) and its deep learning version idTracker.ai (Romero-Ferrero et al., 2019) make tracking unmarked targets possible. In more complex situations, researchers have to extract detailed pose information of the targets (Khan et al., 2012; Guo et al., 2015; Ota et al., 2015), which raises the difficulty of tracking. Representative methods, DLC (Mathis et al., 2018), LEAP (Pereira et al., 2019), and DeepPoseKit (Graving et al., 2019), display exemplary performance in pose estimation by utilizing the outstanding feature extraction ability of deep-learning network. However, the amount of tracking individuals is still limited due to the increased computation time. Moreover, it requires exhaustive annotating efforts to establish a training dataset with the growing individuals (Graving et al., 2019). Individual tracking and pose tracking are closely related. On the one hand, tracking an individual or a local body is always performed to crop active areas of the object to achieve better pose tracking. On the other hand, pose tracking can be regarded as high dimensional individual tracking in some ways, tracking key points of animals (Dell et al., 2014). Therefore, accurate tracking is the foundation of behavioral analysis, and this is the theoretical basis for SNAP-Tracker to be applied in broader tasks.

Overall, SNAP-Tracker perfectly achieves a balance between applicability and accuracy. The pretrained deep Siamese network makes SNAP-Tracker track the object accurately by comparing the similarity between the template and the target. The model-free tracking strategy equips SNAP-Tracker with broader applicability demonstrated by the experiments above in this article. Strictly speaking, the problem of manual annotation is not thoroughly solved, but the most attractive characteristic of SNAP-Tracker is that it requires only one annotation to start the tracking procedure. Users can correct accidental tracking failures by hands in regular mode or by the detection module in the “tracking with detection” mode. For the convenience of users, we designed the detection module is in a close loop, in which the tracking module offers elementary results as training data to the detection module, and the latter can help increase the accuracy of tracking. There is still some weakness of the SNAP-Tracker that should be solved to improve further the usability and accuracy of SNAP-Tracker, such as the setting of optimal hyperparameters (Dong et al., 2021) and the tracking failures when the target is occluded (Dong et al., 2017). We have considered some of these in the subsequent improvement of SNAP-Tracker.

## Conclusion

In conclusion, we provide a tracking method in a model-free fashion. Users can easily apply it to various tasks without heavy data annotations. We hope that our tool can lower the barrier to using deep learning methods in animal behavioral analysis and help solve practical tracking problems in related fields.

## Data Availability Statement

The datasets presented in this study can be found in online repositories. The names of the repository/repositories and accession number(s) can be found below: https://drive.google.com/file/d/1k0w_lgIBd5xIY0f63J8VfuccvHZ7spsD/view?usp=sharing.

## Author Contributions

LS, WW, KD, YT, and LM conceived and designed the experiments. LS wrote the code and annotated the dataset. LS and KS performed the experiments. LS, WW, KS, XL, and KD analyzed the results. LS, WW, and KS prepared the figures. LS, WW, YT, and LM wrote and revised the draft. All authors provided comments and approved the manuscript.

## Conflict of Interest

The authors declare that the research was conducted in the absence of any commercial or financial relationships that could be construed as a potential conflict of interest.

## Publisher’s Note

All claims expressed in this article are solely those of the authors and do not necessarily represent those of their affiliated organizations, or those of the publisher, the editors and the reviewers. Any product that may be evaluated in this article, or claim that may be made by its manufacturer, is not guaranteed or endorsed by the publisher.
