# Supplementary figures and images for: Siamese Network-Based All-Purpose-Tracker, a Model-Free Deep Learning Tool for Animal Behavioral Tracking
Source: Front Behav Neurosci. 2022 Mar 4;16:759943. doi: 10.3389/fnbeh.2022.759943 (PMC8931526; doi:10.3389/fnbeh.2022.759943)

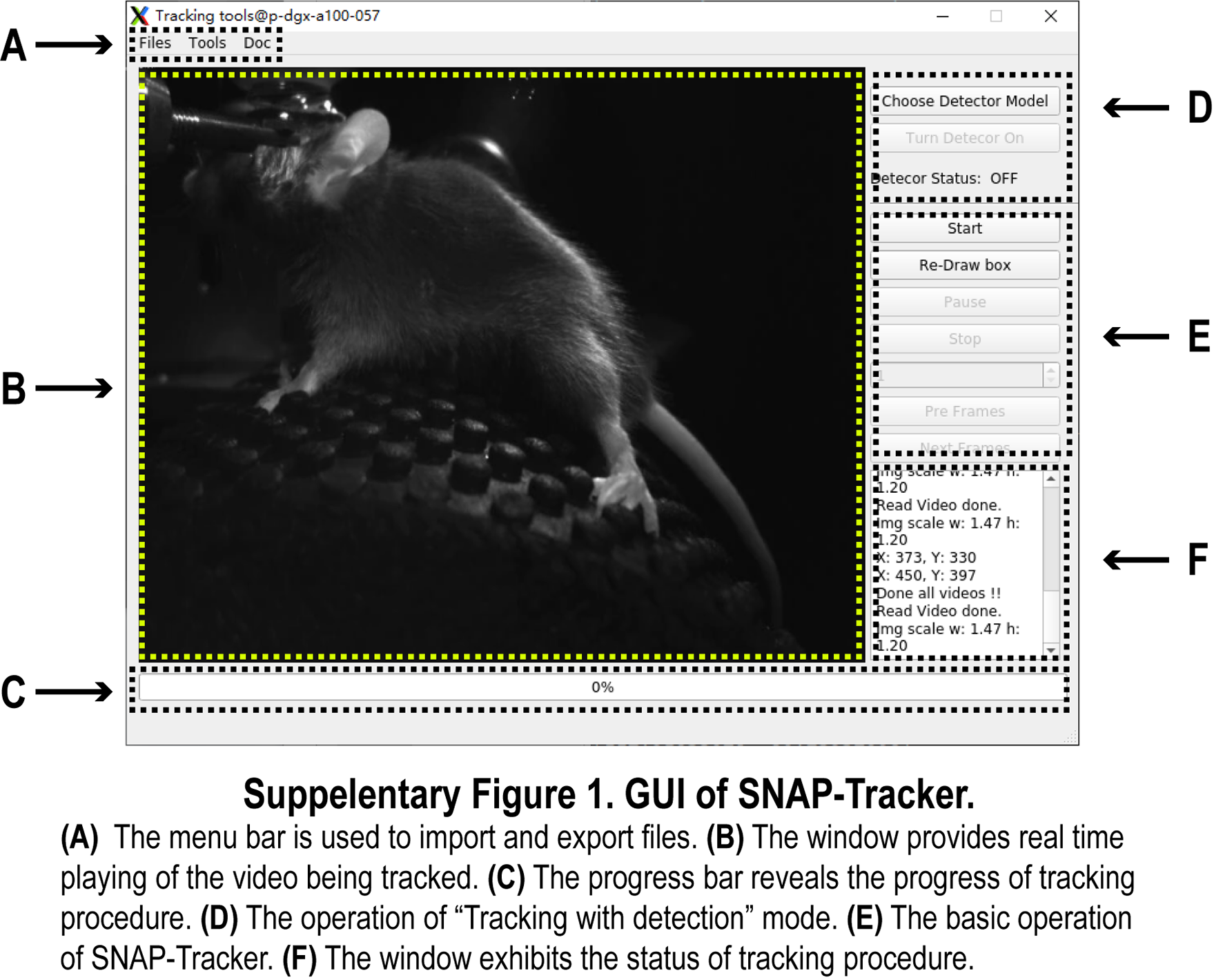

Supplement: Supplementary file 1 [file Image_1.TIF]

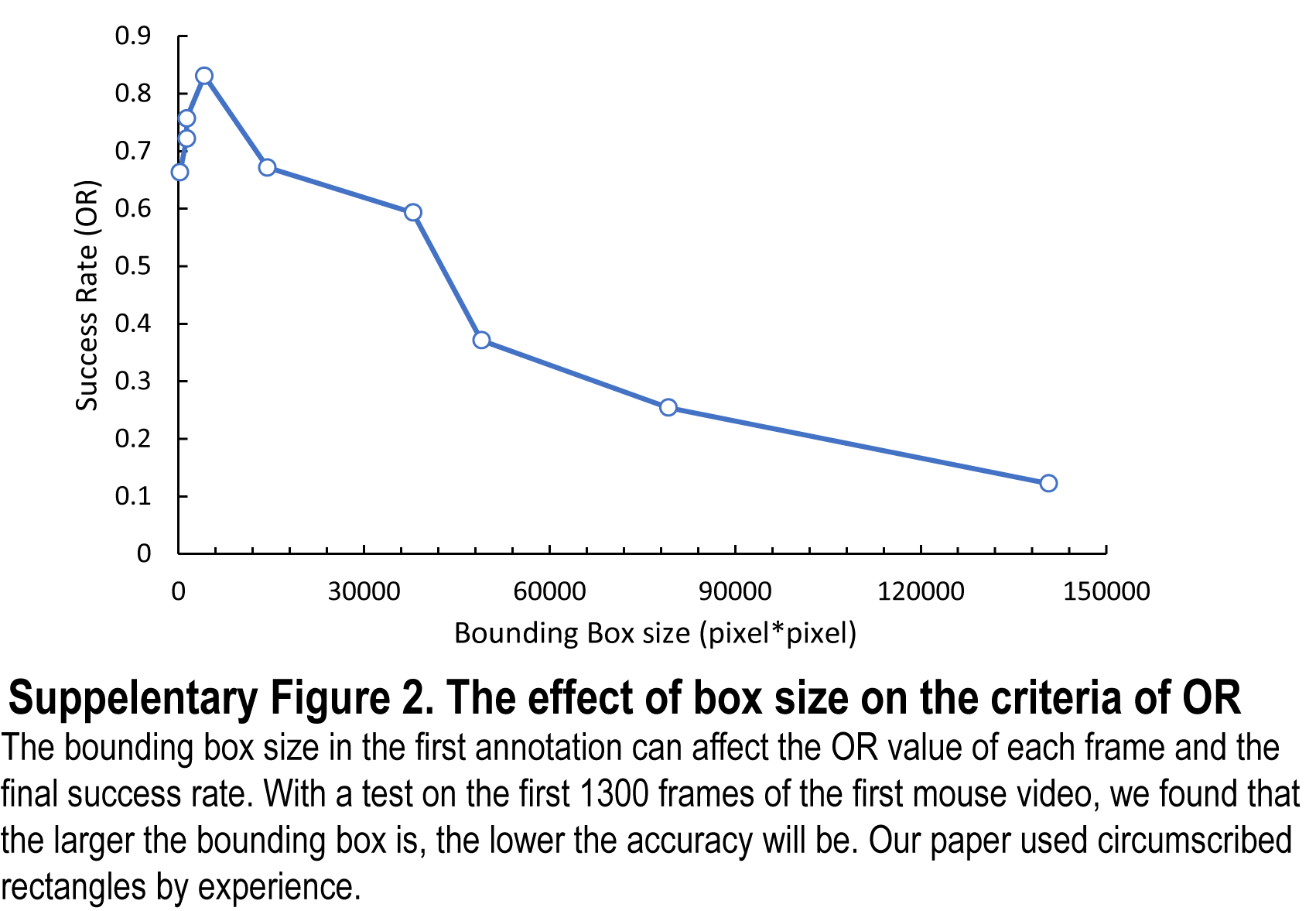

Supplement: Supplementary file 2 [file Image_2.TIF]

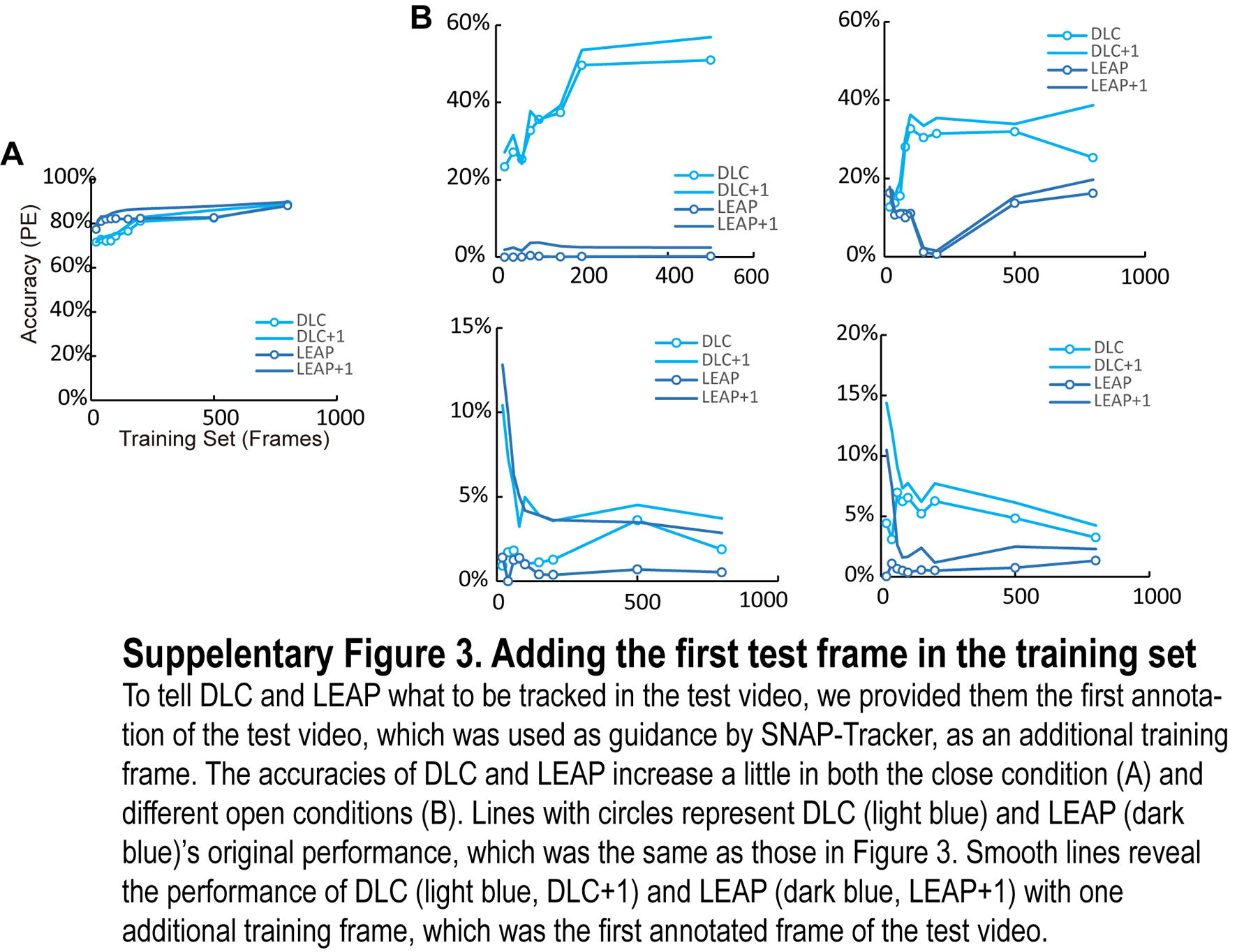

Supplement: Supplementary file 3 [file Image_3.TIF]

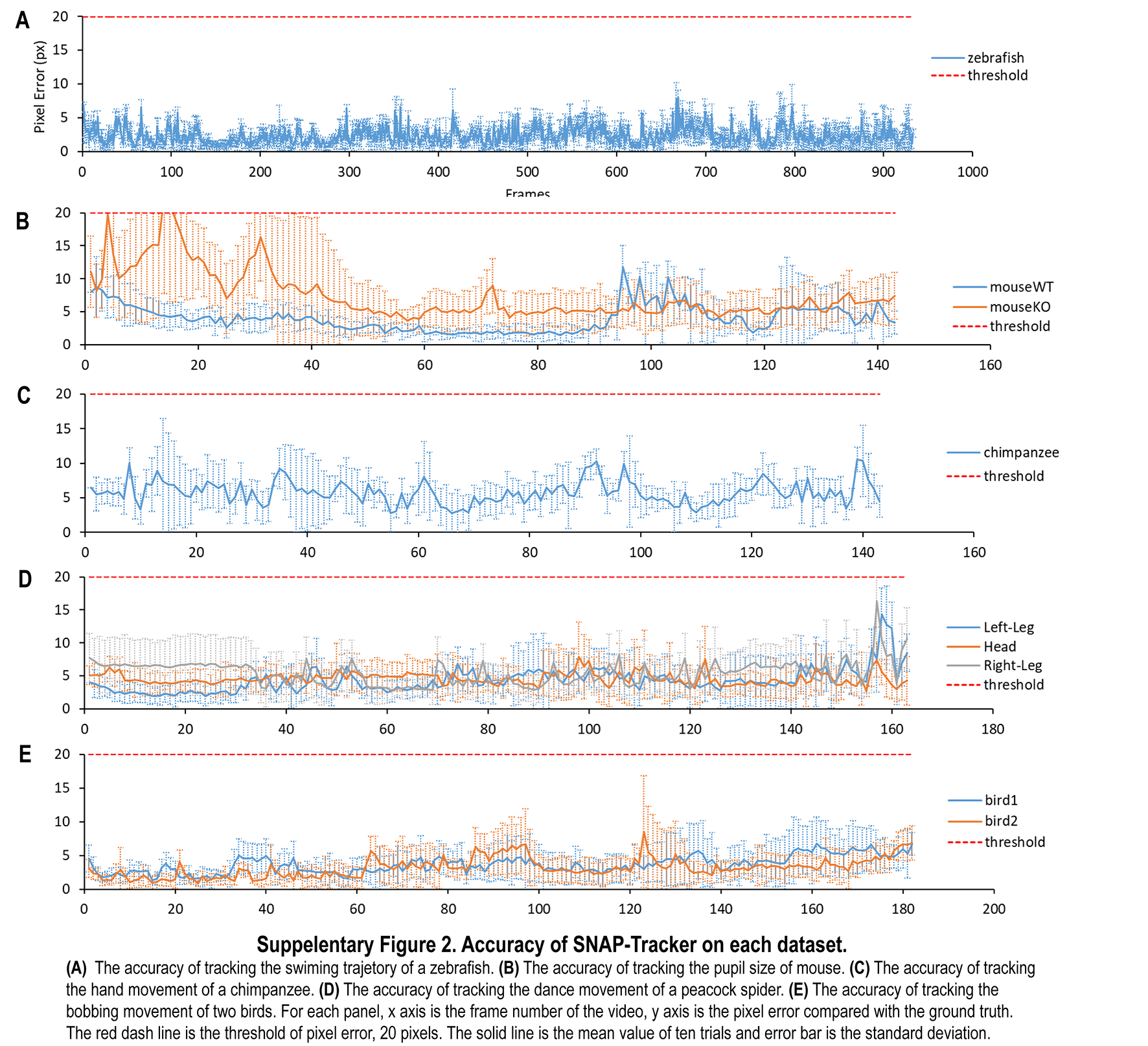

Supplement: Supplementary file 4 [file Image_4.TIF]
